# Exploring *IGF-1* Gene Polymorphisms in Diverse Saudi Arabian Dromedary Camel Breeds

**DOI:** 10.3390/cimb48040383

**Published:** 2026-04-07

**Authors:** Saleh M. Albarrak, Fahad. A. Alshanbari, Ali Almedaid, Mohammed Albugshi

**Affiliations:** 1 Department of Pathology and Laboratory Diagnosis, College of Veterinary Medicine, Qassim University, Buraydah City 52571, Saudi Arabia; 2 Department of Medial Biosciences, College of Veterinary Medicine, Qassim University, Buraydah City 52571, Saudi Arabia; 3 College of Veterinary Medicine, Qassim University, Buraydah City 52571, Saudi Arabia; 431107445@qu.edu.sa (A.A.); 431107587@qu.edu.sa (M.A.)

**Keywords:** genetic variability, dromedary camel, *IGF-1* polymorphism, selective breeding

## Abstract

The insulin-like growth factor 1 (*IGF-1*) gene plays a key role in growth and production traits in livestock. Limited information is available regarding its genetic polymorphisms in Saudi camel breeds. This study aimed to investigate genetic variation in the *IGF-1* gene among Saudi camel breeds to provide baseline genetic information for future association studies. A total of 176 camels representing six Saudi breeds were sampled. DNA was extracted and Polymerase chain reaction (PCR) amplification and Sanger sequencing were applied to detect *IGF-1* polymorphisms. Genotype and allele frequencies were calculated across breeds, and statistical comparisons were performed based on proportional distributions to account for unequal sample sizes. Two single-nucleotide polymorphisms (SNPs) were identified: c.365G>A in exon 3 and c.435C>T in exon 5. The exon 3 variant resulted in a missense mutation (p. Arg122His) but was detected in heterozygous form in only one camel, and subsequent screening of 109 additional samples confirmed its rarity. The exon 5 variant was synonymous in isoform X1 and located in the 3′ untranslated region of isoform X2. Sequencing of 176 camels revealed that c.435C>T was highly polymorphic across the examined breeds. Significant differences in genotype frequencies were observed within and among breeds (*p* < 0.001). The CT genotype predominated in Waddah (60%), Shageh (48%), and Sofor (60%), significantly exceeding CC and TT frequencies (*p* < 0.001). In Majaheem and Saheli, CT (47%) and TT (45%) were nearly equal and both significantly higher than CC (*p* < 0.001). Shaele exhibited a distinct pattern, with TT being most frequent (57%), significantly higher than CC (7%, *p* < 0.001) and CT (36%, *p* < 0.01). These findings indicate directional selection favoring the C allele in the Waddah and Shageh breeds, whereas the T allele is favored in the remaining breeds. This study provides the first baseline characterization of *IGF-1* polymorphisms among Saudi camel breeds. Although no phenotypic associations were assessed, the results offer a foundation for future research examining relationships between *IGF-1* variants and economically important traits.

## 1. Introduction

The camel occupies a distinctive position in Arab culture, valued both for its economic role as a source of food and its symbolic representation of strength and resilience. Its significance spans centuries, encompassing historical, religious, and contemporary contexts, thereby forming an integral component of Arab heritage. Saudi Arabia hosts several phenotypically distinct breeds of Camelus dromedarius, including Waddah, Shageh, Shaele, Sofor, Majaheem, and Saheli, which have been previously characterized [1,2]. Evaluation of morphological traits such as body weight, height, length, and width is essential for determining growth performance, meat yield, and overall productivity [3]. Furthermore, the selection of genotypes associated with superior growth and meat production traits is critical for enhancing the camel’s contribution to the agricultural economy [4,5].

Exploring genomic regions associated with morphometric traits in camels offers critical insights into their adaptive and productive characteristics [5,6,7,8]. Among these regions, the insulin-like growth factor-1 (*IGF-1*) gene plays a pivotal role in regulating growth and development, influencing fetal and postnatal growth, cell differentiation, embryogenesis, and metabolic processes [5,6]. The *IGF-1* gene encodes a ~153-amino acid protein with a molecular weight of approximately 7.5 kDa [9] and, in camelids, consists of five exons and four introns [6]. While polymorphisms in *IGF-1* have been extensively studied in various species such as cattle, buffalo, sheep, goats, and chicken, research in camels remains limited [10,11,12,13,14]. Previous studies in Pakistani Marecha camels have reported four polymorphic sites in the *IGF-1* gene, including a T→C substitution in exon 5 that results in a cysteine-to-arginine change, highlighting the polymorphic nature of this gene [6].

Associations between *IGF-1* gene variants and growth traits have been well documented in other livestock species. For example, a C-512T SNP in the promoter region was linked to growth performance in Angus cattle [15], while similar correlations between *IGF-1* polymorphisms and body weight have been observed in Nelore, Canchim, and other breeds [16]. Additionally, an *IGF-1*/SnaBI SNP in the promoter region was associated with growth traits in Charolais cattle [17]. A recent study by Dakheel et al. (2025) reported that Iraqi camels exhibited three *IGF-1* exon 5 genotypes AA, Aa, and aa with the heterozygous Aa genotype showing superior weight gain from birth to six months of age [18]. These findings underscore the functional significance of *IGF-1* in influencing growth patterns across species and suggest that identifying and characterizing its variants in camels could provide valuable markers for genetic improvement programs aimed at enhancing productivity and adaptability.

Although the *IGF-1* gene is a key regulator of growth and developmental processes, its polymorphic variations in Saudi dromedary camels and their potential influence on morphometric traits remain largely uncharacterized. This lack of genetic information represents a significant research gap, particularly given the economic and biological importance of these breeds. Therefore, the objective of this study is to address this knowledge gap by characterizing *IGF-1* gene polymorphisms in commonly reared Saudi camel breeds and provide foundational data for future association studies. Polymerase chain reaction (PCR) amplification and Sanger sequencing were employed to identify genetic variants

## 2. Materials and Methods

### 2.1. Ethical Approval

Blood samples were collected from camels using exclusively non-invasive procedures that caused no harm or distress to the animals. All samplings were performed by certified veterinarians to ensure animal welfare and adherence to best professional practices. The Blood sampling procedure was conducted in accordance with institutional guidelines and was approved by the Animal Care and Use Committee (ACUC) at Qassim University (Approval No. 26-6-11; Approval date 4 February 2026).

### 2.2. Animals

The study involved 176 dromedary camels, comprising six breeds: Waddah (*n* = 58), Shageh (*n* = 29), Shaela (*n* = 28), Sofor (*n* = 20), Majaheem (*n* = 30), and Sahile (*n* = 11), and included animals of mixed sexes ranging in age from 6 months to 20 years. All samples were collected from animals located in the Qassim region (central region) except for the Sahile camels, which were sampled in the western region. There are no differences between the two regions in terms of climate, vegetation, or management practices. Camels grazed in open rangeland from sunrise until midday, after which they were brought into the farm and provided supplemental grain. They received 3–4 kg/day of dry hay (*Chloris gayana* or wheat straw) and barley-based concentrate. Unequal sample sizes among breeds reflect natural differences in herd distribution and field accessibility. Breeds such as Saheli and Sofor are maintained in small, dispersed herds, limiting the number of animals available for sampling. The representativeness of smaller samples (e.g., Saheli, *n* = 11) was supported by multi-herd sampling, avoidance of first-degree relatives, and allele-detection probabilities: using *P* = 1 − (1 − p)^2^*^n^*, alleles at 10% and 20% frequencies were detectable at ~90% and ~99% probability, indicating that the sampling strategy was adequate for capturing common and moderate-frequency *IGF-1* variants.

### 2.3. Blood Collection

Blood samples were collected from the jugular vein into EDTA-containing tubes and stored at 4 °C until processing.

### 2.4. DNA Isolation

Genomic DNA was extracted from peripheral blood leukocytes using a Qiagen Blood Extraction Kit (Cat#: 51104; QIAGEN Sciences, Germantown, MD, USA) in accordance with the manufacturer’s protocol. DNA concentration and purity were evaluated using a NanoDrop spectrophotometer (Thermo Fisher Scientific, Waltham, MA, USA), and integrity was confirmed by gel electrophoresis on a 1% agarose gel.

### 2.5. Primers Design

**Figure 1:**
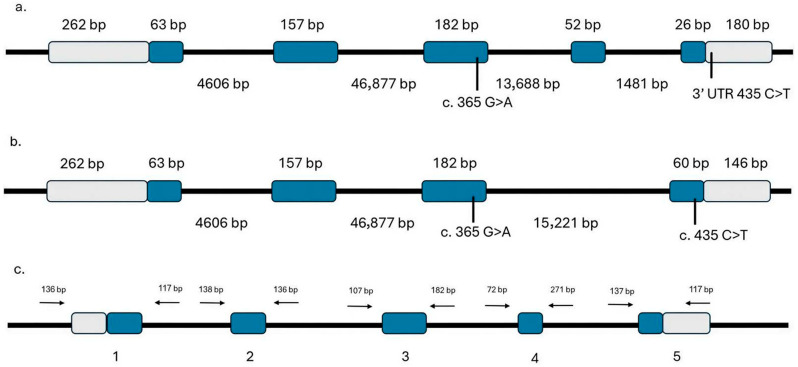
Schematic representation of the *IGF-1* gene structure for its two isoforms and primer binding sites. (**a**) Isoform X1 genomic arrangement showing five coding exons (blue boxes), four introns (black lines), and the 5′ and 3′ untranslated regions (gray boxes). Exon and UTR lengths (bp) are indicated above each box, and intron lengths are shown below the line. (**b**) Isoform X2 genomic arrangement with four coding exons (blue boxes) and three introns (black lines). The 5′ and 3′ UTRs (gray boxes) are shown, with the 3′ UTR differing in length from Isoform X1. Single-nucleotide polymorphisms (SNPs) within the *IGF-1* gene are depicted as vertical black lines with their corresponding positions. (**c**) Primer binding sites mapped across the genomic structure of *IGF-1*. Coding exons are represented in dark blue and untranslated regions in light gray. Forward primers are indicated by right-facing arrows, and reverse primers by left-facing arrows. Numbers above each arrow denote the distance (bp) from the nearest exon boundary.

The *IGF-1* gene is located on chromosome 11 and contains five exons (Gene ID: 105097468), spanning 67,520 nucleotides. Two isoforms of the IGF-1 genomic region have been reported: isoform X1 (XP_010988322.1) comprises five exons, whereas isoform X2 (XP_010988323.1) contains four exons (Figure 1). The genomic sequence was obtained from the dromedary camel reference genome mCamDro1.pat (GCF_036321535.1) available in the national center for biotechnology information (NCBI) Gene database (https://www.ncbi.nlm.nih.gov/gene/?term=105097468, accessed on 1 April 2026). The primer pair was designed based on the *IGF-1* isoform X1 sequence; however, isoform X2 differs only by the absence of exon 4, which does not affect the primer binding sites.

Primers were designed using Primer3 software (https://primer3.ut.ee/, version 4.1.0, accessed on 1 March 2025) to amplify the coding regions, including exon–intron boundaries, the 5′ untranslated region (UTR), and the 3′ UTR (Table 1) [19]. Repetitive elements within the target sequences were identified using RepeatMasker (https://www.repeatmasker.org/cgi-bin/WEBRepeatMasker, version 4.0.9, accessed on 1 March 2025). The primer sites for the amplicons are illustrated in Figure 1c.

### 2.6. PCR Amplification and Sanger Sequencing

PCR amplification was carried out in 20 μL reactions containing 1 unit of DreamTaq DNA polymerase master mix (Cat#: K1071, Thermo Fisher Scientific, Waltham, MA, USA), 50 ng of genomic DNA, 10 pmol of each primer, and nuclease-free water. The thermal cycling protocol consisted of an initial denaturation at 95 °C for 3 min, followed by 35 cycles of denaturation at 95 °C for 30 s, annealing at ~55 °C for 30 s, and extension at 72 °C for 1 min, with a final extension at 72 °C for 10 min. PCR products were purified using ExoSAP-IT™ Express PCR Product Cleanup (Cat#: 75001.1.ML, Thermo Fisher Scientific, Waltham, MA, USA) and subjected to Sanger sequencing with the BigDye^®^ Terminator v3.1 Cycle Sequencing Kit (Cat# 4337455, Applied Biosystems, Carlsbad, CA, USA) on an ABI PRISM 3730XL Analyzer (96-capillary format) at Macrogen Inc. (Seoul, Republic of Korea). All five exons of the *IGF-1* gene were sequenced across the examined breeds (*n* = 4 animals/breed). To validate associations between identified variants and the examined breeds, total of 110 samples for exon 3 and 176 samples for exon 5 were sequenced.

### 2.7. Sequence Assessment

Sequence quality was assessed, trimmed, assembled, and analyzed for polymorphisms using Sequencher v5.2.4 (Gene Codes Corporation, Ann Arbor, MI, USA). The *IGF-1* mRNA reference sequence (XM_010990021.3) from NCBI was used to identify variations within the open reading frame (ORF). Observed sequences were aligned to the reference to determine positional information for subsequent analyses. Protein translation and detection of amino acid changes were performed using ExPASy tools (https://www.expasy.org/, version 3.0, accessed on 10 March 2026), while multiple sequence alignment was conducted with CLUSTALW (https://www.genome.jp/tools-bin/clustalw, version 2.1, accessed on 10 June 2025) [20]. Comparative analysis of *IGF-1* protein sequences across mammalian species was performed using the BLASTP tool (https://blast.ncbi.nlm.nih.gov/Blast.cgi?PROGRAM=blastp&PAGE_TYPE=BlastSearch&LINK_LOC=blasthome, version 2.16.0+, accessed on 10 June 2025) available at NCBI. The functional impact of missense variants was evaluated using PolyPhen-2, which predicts the potential deleterious effects of amino acid substitutions on protein structure and function [21]. MicroRNA Database (miRDB, https://mirdb.org/, accessed on 8 March 2026) was used to predict microRNA binding sites in the 3′UTR of *IGF-1* [22].

### 2.8. Statistical Analysis

Data analysis was performed using GraphPad Prism software (version 9.2, La Jolla, CA, USA). Differences in genotype frequencies within and among camel breeds were evaluated using the Chi-square test and Fisher’s Exact Test, as appropriate. Results are reported as χ^2^ values with corresponding *p*-values, and statistical significance was set at *p* < 0.05 for all comparisons. Allele frequencies, observed heterozygosity (Ho), expected heterozygosity (He), and polymorphism information content (PIC) were calculated using established population-genetic formulas for single-locus metrics. Hardy–Weinberg equilibrium (HWE) was assessed with a chi-square (χ^2^) test. All statistical procedures were independently reviewed by a population-genetics specialist to confirm their appropriateness for the dataset and sample size.

## 3. Results

### 3.1. IGF-1 Gene Genomic Structure

In dromedary camels, the *IGF-1* gene is expressed in two isoforms, X1 and X2. Both isoforms share the same basic genomic structure but are generated through alternative splicing. They have identical genomic regions for exons 1, 2, and 3, while exon 4 is present only in isoform X1. Additionally, exon 5 is included in both isoforms but originates from different genomic regions in X1 and X2 (Figure 1a,b, and Table 2).

### 3.2. IGF-1 Gene Variants

**Figure 2:**
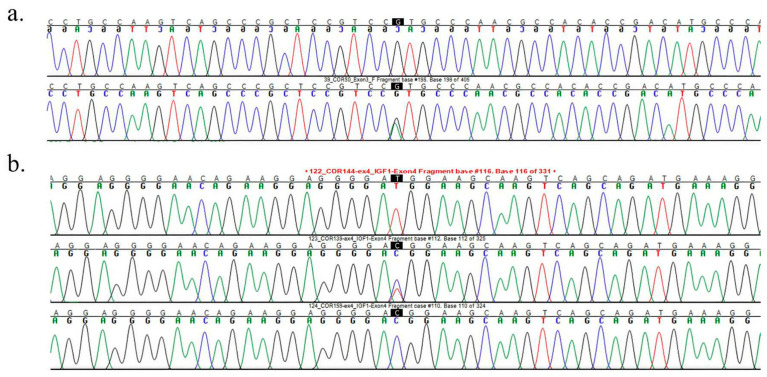
(**a**) Chromatogram showing a point mutation in the sequenced exon 3 c.365 G>A fragment. Electropherogram traces display nucleotide peaks for both forward and reverse strands. The highlighted position indicates a single-nucleotide variation (G→A) within the analyzed region. Colored peaks represent individual bases (blue = C, red = T, green = A, black = G), and the sequence context is shown above the chromatogram. (**b**) Chromatogram showing a point mutation in the sequenced exon 5 c.435 C>T fragment. Electropherogram traces display nucleotide peaks for both forward and reverse strands. The highlighted position indicates a single-nucleotide variation (C→T) within the analyzed region. Colored peaks represent individual bases (blue = C, red = T, green = A, black = G), and the sequence context is shown above the chromatogram.

Two single-nucleotide polymorphisms (SNPs) were identified during the initial variant screening: one in exon 3 (c.365G>A) and another (c.435C>T) lies within exon 5 of isoform X1 and corresponds to the 3′ UTR of isoform X2 (Figure 2a and Figure 2b, respectively). Based on the reference genome, the c.365GG and c.435CC genotypes represent the wild-type forms. No variations were detected in exons 1, 2, or 4. The exon 3 variant (c.365G>A) resulted in a missense mutation, substituting arginine with histidine at position 122 (p.Arg122His). However, this variant was observed in heterozygous form in only one camel. Subsequent sequencing of an additional 109 camels did not reveal the A allele, indicating that this variant is extremely rare. Using PolyPhen-2 to assess the functional impact of the amino acid substitution, the analysis predicted that the mutation is probably damaging with a score of 1.000, indicating a high likelihood of deleterious effects on the protein.

The exon 5 variant (c.435C>T) represented a synonymous mutation in isoform X1 but was in the 3′ untranslated region (UTR) of isoform X2 (Figure 1). MicroRNA Database (miRDB) did not predict any microRNA (miRNA) binding sites specifically within the 3′ UTR. Table 3 summarizes the distribution of observed genotypes across the studied camel breeds. Significant variation in genotype frequencies was detected within all breeds (*p* < 0.001). In Waddah, Shageh, and Sofor breeds, the CT genotype was predominant, accounting for 60%, 48%, and 60% of individuals, respectively. This frequency was significantly higher than that of CC and TT genotypes (*p* < 0.001). In Majaheem and Saheli breeds, CT (47%) and TT (45%) genotypes exhibited nearly equal representation, both significantly exceeding the CC genotype (*p* < 0.001). Shaele camels showed a distinct pattern, with the TT genotype being the most common (57%), significantly higher frequency than the CC and CT genotypes (*p* < 0.01).

These findings indicate breed-specific differences in genotype distribution, suggesting potential selective pressures influencing allele frequencies.

Significant differences in observed genotype frequencies were detected among the examined camel breeds (Table 3). The CC genotype was most frequent in Shageh (24%) and Waddah (19%), with both breeds showing significantly higher CC frequencies compared to Shaele (7%), Sofor (5%), Majaheem (7%), and Saheli (10%) (*p* < 0.001). For the CT genotype, Waddah and Sofor exhibited the highest frequencies (60% each). These values did not differ significantly from those observed in Shageh, Majaheem, and Saheli (*p* > 0.05). In contrast, Shaele displayed the lowest CT frequency (36%), which was significantly lower compared to Waddah and Sofor (*p* < 0.01). This pattern suggests that while CT is generally prevalent across most breeds, Shaele shows a marked reduction, indicating possible breed-specific genetic variation.

Analysis of TT genotype frequencies revealed a clear dominance in Shaele camels, where it accounted for 57% of individuals. This proportion was significantly higher compared to Sofor (35%, *p* < 0.01), Shageh (28%, *p* < 0.01), and Waddah (21%, *p* < 0.001). However, no significant differences were observed when comparing Shaele to Majaheem (47%) and Saheli (45%) (*p* > 0.05). These findings indicate that the TT genotype is strongly favored in Shaele, while its prevalence in other breeds varies, suggesting potential breed-specific genetic patterns or selective pressures influencing allele distribution.

As shown in Table 4, genotype frequencies vary significantly among the examined breeds (*p* < 0.043). The T allele (q) is more common in all populations except for Shageh and Waddah, where C and T are almost balanced. Observed heterozygosity (Ho) ranges from 0.357 (Shaele) to 0.603 (Waddah). Most populations show Ho > He, indicating possible heterozygote excess which could result from mixing populations or balancing selection. The highest polymorphic information content (PIC) values are in Waddah (0.2499) and Shageh (0.2494), indicating slightly more informative polymorphism. All PIC values are <0.25, which is typical for SNP markers and indicates low to moderate informativeness. No population shows statistically significant deviation from Hardy–Weinberg Equilibrium (HWE).

### 3.3. Comparative Multiple Sequence Alignment of IGF-1 Protein in Dromedary Camel and Other Mammalian Species Orthologs

**Figure 3:**
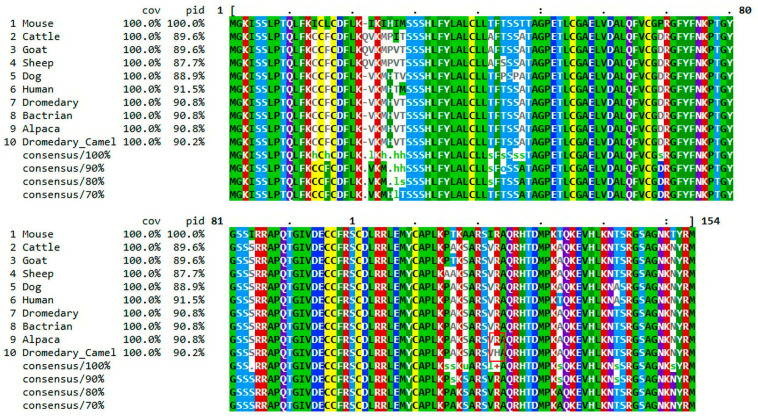
Multiple sequence alignment of IGF-1 protein across mammalian species. Alignment shows IGF-1 amino acid sequences from ten species, including mice, cattle, goat, sheep, dog, human, dromedary camel, Bactrian camel, and alpaca. Coverage (cov) and percent identity (pid) values are listed for each species relative to the reference sequence. Conserved residues are highlighted by color coding, with consensus sequences displayed at different thresholds (100%, 90%, 80%, and 70%), and a change in color within a column indicates a lack of residue conservation at that position. The alignment demonstrates strong conservation among camelids (100% identity) and high similarity with human and dog sequences (>90%), while ruminants exhibit lower identity. Positions 1–80 and 81–154 represent two segments of the IGF-1 protein. The red box indicates the position of the R-to-H amino acid substitution at residue 122, which arises from the missense mutation located in exon 3.

*IGF-1* is highly conserved among camelids (dromedary, Bactrian camel, and alpaca), exhibiting 100% sequence identity. Furthermore, the IGF-1 amino acid sequences are highly conserved across many mammalian species including cattle, sheep, goat, dogs, mice and humans (sequence identity ~96%). Overall, the consensus sequence highlights the conserved core structure of IGF-1 across species (Figure 3). As shown in Figure 3, the R is highly conserved across mammalian species. However, the red box in Figure 3 indicates R-to-H amino acid substitution at position 122, which arises from the missense mutation located in exon 3.

## 4. Discussion

This study provides novel insights into the genetic variability of the *IGF-1* gene in Saudi dromedary camels and establishes a valuable foundation dataset for future association and functional studies. Two single-nucleotide polymorphisms (SNPs) were identified: a missense variant in exon 3 (c.365G>A; p.Arg122His) and a synonymous variant in exon 5 (c.435C>T). The exon 3 missense variant represents a novel polymorphism which was detected in only one heterozygous individual. Its absence in the extended sample set indicates that this mutation exists at extremely low population frequency and is unlikely to exert a significant effect on population-level phenotypic variation. This pattern aligns with earlier studies in camel and livestock species, where *IGF-1* coding-region polymorphisms tend to be rare or absent. For example, Egyptian Maghrabi camels exhibited no coding variation, and Marecha camels showed polymorphisms predominantly in non-coding regions rather than exonic sites [6,23]. The rarity of this mutation suggests that it may represent a spontaneous mutation or lineage-specific event without broader adaptive significance, consistent with previous reports of sporadic IGF-1 coding variants in other mammals [24,25].

In contrast, exon 5 SNP (c.435C>T) exhibited moderate polymorphism across all examined breeds, making it the principal contributor to genetic variation in this study. Although this substitution is synonymous in isoform X1 and located in the 3′ untranslated region (UTR) of isoform X2, its widespread distribution raises the possibility of regulatory implications. UTR substitutions can influence mRNA stability and translational efficiency, or RNA-binding protein interactions across mammalian species [26,27]. While the miRDB analysis did not reveal predicted miRNA binding sites, the involvement of other post-transcriptional mechanisms cannot be excluded, underscoring the need for future functional validation.

Similar patterns have been documented internationally. In Pakistani Marecha camels, non-coding *IGF-1* polymorphisms—including a T→C mutation in exon 5 were—associated with growth traits, whereas African populations such as Egyptian Maghrabi camels showed extremely limited *IGF-1* coding variability but did exhibit GH/IGF-axis polymorphisms linked to growth performance [6,23]. More recently, Dakheel et al. (2025) reported three distinct *IGF-1* exon 5 genotypes in Iraqi camels, each associating with significant differences in weight gain from birth to one year of age [18]. While these findings underscore the biological relevance of the *IGF-1* regulatory region in other camel populations, it is important to note that the present study did not evaluate phenotypic traits and therefore cannot infer functional consequences of the detected variants. Nonetheless, the genetic variations observed among the breeds analyzed in the present study aligned with previous reports, suggesting that *IGF-1* diversity in camels occurs predominantly within coding regions rather than regulatory regions [6,18]. This pattern indicates notable genetic differentiation, which may reflect underlying selective pressures as well as breed- specific management practices.

The predominance of the CT genotype in Waddah, Shageh, and Sofor suggests that heterozygosity at this locus could confer adaptive or production-related advantages, such as improved growth performance, metabolic efficiency, or resilience to environmental stress, traits often favored by breeders. This aligns with extensive livestock research showing heterozygous individuals often exhibit superior fitness compared with homozygotes, a phenomenon commonly attributed to heterosis, which can enhance growth, immunity, and overall physiological stability across species [28]. Although the present findings indicate that the CT genotype may be associated with advantageous phenotypic outcomes, direct functional evidence in camels remains limited, underscoring the need for targeted genotype–phenotype studies to clarify its role and potential value in future marker-assisted selection programs.

In contrast, the Shaele breed displayed a distinct pattern, with the TT genotype occurring at a markedly higher frequency than the other genotypes. This pronounced dominance may indicate directional selection favoring the T allele within this population, possibly due to its association with breed-specific adaptive traits, such as adaptation to harsh environmental conditions or unique physiological characteristics. In African and Central Asian camel populations, available *IGF-1* studies report either balanced or relatively low TT frequencies [6,23]. Therefore, the strong TT skew observed **i**n the Shaele breed may reflect localized directional selection or breed-specific adaptive pressures not previously documented in other regions.

By comparison, the relatively balanced frequencies of CT and TT genotypes in Majaheem and Saheli suggest a more diverse genetic structure, potentially reflecting weaker selective pressures or more heterogeneous breeding histories. However, it is important to note that the interpretation of genotype distribution in the Saheli breed should be approached with caution due to its small sample size (*n* = 11), which may limit the reliability and generalizability of these observations.

The consistently lower frequency of the CC genotype across most breeds, except Shageh and Waddah, may imply that the C allele is either less advantageous or subject to negative selection in certain populations. However, its maintenance at moderate levels in some breeds suggests that it is not entirely disadvantageous and may contribute meaningfully to overall genetic variability. Comparable patterns have been reported in cattle and sheep, where *IGF-1* polymorphisms in regulatory regions showed variable associations with growth traits depending on population size and environmental factors [17,29]. Taken together, the observations of the current study underscore the influence of breed-specific selection histories on *IGF-1* genetic variation and highlight the need for further investigations with larger sample sizes and additional phenotypic data to clarify potential functional consequences.

Comparative sequence analysis revealed that IGF-1 protein is highly conserved among camelids, with complete identity across dromedary, Bactrian camel, and alpaca sequences. This conservation underscores the essential role of IGF-1 in growth and metabolic regulation within Camelidae, consistent with previous studies highlighting IGF-1’s evolutionary stability across vertebrates [30,31]. The IGF-1 amino acid sequences are highly conserved across mammalian species. The overall conservation of the core IGF-1 structure across mammals highlights its evolutionary importance in growth regulation, while observed interspecies variability may contribute to differences in growth patterns and metabolic strategies [32].

A key limitation of this study is the unequal sample sizes among breeds, which may reduce the ability to detect rare *IGF-1* genotypes in smaller groups such as Saheli. Consequently, the results are interpreted descriptively rather than as formal inferential conclusions. Because the primary aim was to document existing *IGF-1* polymorphisms rather than assess population-level evolutionary or selection processes, the findings should be regarded as an initial survey of genetic variation across breeds. Future studies with larger and more balanced sample sizes will be essential for validating the allele-frequency patterns observed here and enabling more robust statistical comparisons among breeds.

## 5. Conclusions

The findings of the current investigation provide the first comprehensive assessment of *IGF-1* genetic variability in Saudi dromedary camels. The observed patterns may have practical implications for genetic improvement programs, particularly if specific *IGF-1* genotypes are later found to be linked to economically important traits. Despite these advances, several limitations warrant consideration. Future research should include larger and more geographically diverse sampling to capture population-level variation more accurately and to minimize potential sampling bias. Moreover, functional analyses are needed to elucidate the biological consequences of the identified variant and to determine whether it contributes directly to phenotypic differences among camel breeds. Finally, we recommend expanding future investigations into additional regional populations to enable broader comparative analyses and to strengthen our understanding of the evolutionary and breeding forces shaping *IGF-1* variability in camels.

## Figures and Tables

**Table 1:** List of primer sets to amplify and sequence the genomic region of *IGF-1* gene.

No.	Oligo Name	5′-Oligo Seq-3′ Forward Primer	5′-Oligo Seq-3′ Reverse Primer	Product Size	Aneal Tm (°C)
1	*IGF-1* exon 1	TAATGTCTGCTCACCCTGTCA	AACTCTCAGTGCCGAAACAAT	592 base pair	54.3
2	*IGF-1* exon 2	ACCTCCTGTTGCACTTCTGAG	GCTGAAACACTAGGCTCACTT	432 base pair	55.3
3	*IGF-1* exon 3	AAATGTGTGGGTTGACAAGGT	CCAGAGCAGCAGAGGGAATAA	472 base pair	55.6
4	*IGF-1* exon 4	CAAAGGCTCTGTCTTCTGGGA	CTTCTCCTTCTGTCACATGCG	395 base pair	56.6
5	*IGF-1* exon 5	AAAACCAGGCCCAAGTTGTTT	TACTTGCGTATTTCACTGGGG	333 base pair	54.7

**Table 2:** *IGF-1* (GeneID: 105097468) isoforms X1 and X2 details based on NCBI data base (https://www.ncbi.nlm.nih.gov/gene/?term=105097468, accessed on 1 April 2026).

*IGF-1* Isoforms	No. of Exons	mRNA ID	Protein ID	Span on NC_087446.1	Aligned Length	CDS Length	Protein Length
Isoform X1	5	XM_010990020.3	XP_010988322.1	67,499 nt	921 nt	480 nt	159 aa
Isoform X2	4	XM_010990021.3	XP_010988323.1	67,520 nt	890 nt	462 nt	153 aa

**Table 3:** Observed genotype frequencies of Exon 5 c.435 C>T for all studied camel breeds.

Phenotype	Observed Genotype Frequency (%)			Total	*: *X*^2^ and *p*-Value
	CC	CT	TT		
Waddah	11 (19%)	35 (60%)	12 (21%)	58	*X*^2^ = 36.92 *p* = 0.0001
Shageh	7 (24%)	14 (48%)	8 (28%)	29	*X*^2^ = 14.88 *p* = 0.0006
Shaele	2 (7%)	10 (36%)	16 (57%)	28	*X*^2^ = 56.73 *p* = 0.0001
Sofor	1 (5%)	12 (60%)	7 (35%)	20	*X*^2^ = 68.25 *p* = 0.0001
Majaheem	2 (7%)	14 (47%)	14 (47%)	30	*X*^2^ = 47.76 *p* = 0.0001
Saheli	1 (10%)	5 (45%)	5 (45%)	11	*X*^2^ = 36.75 *p* = 0.0001
Total	25	96	65	176	
#: *X*^2^ *p*-value	28.03 0.0001	17.26 0.04	37.25 0.0001		

* Significant variations in the observed genotype within breed. #: Significant variation in observed genotype among breeds.

**Table 4:** Allele frequencies and genetic diversity parameters for the examined camel breeds.

Population	N	p(C)	q(T)	Ho	He	PIC	χ^2^ (HWE)	HWE *p*-Value
Waddah	58	0.491	0.509	0.6034	0.4999	0.2499	2.491	0.114
Shageh	29	0.483	0.517	0.4828	0.4994	0.2494	0.0322	0.858
Shaele	28	0.25	0.75	0.3571	0.3750	0.1406	0.0635	0.801
Sofor	20	0.35	0.65	0.6000	0.4550	0.2070	2.031	0.154
Majaheem	30	0.30	0.70	0.4667	0.4200	0.1764	0.370	0.543
Saheli	11	0.318	0.682	0.4545	0.4339	0.1883	0.0249	0.875

N = sample size; p(C) and q(T) = allele frequencies of alleles C and T, respectively; Ho = observed heterozygosity; He = expected heterozygosity according to Hardy–Weinberg expectations; PIC = polymorphic information content; χ^2^ (HWE) = chi-square statistic for deviation from Hardy–Weinberg equilibrium.

## Data Availability

The original contributions presented in this study are included in the article. Further inquiries can be directed to the corresponding authors.

## Abbreviations

The following abbreviations are used in this manuscript:

IGF-1	Insulin-Like Growth Factor 1
TMHMM	TransMembrane Hidden Markov Model
SNPs	Single- nucleotide polymorphisms
NCBI	National Center for Biotechnology Information
UTR	Untranslated Region
